# Comparison of Auxin and Cytokinins Concentrations, and the Structure of Bacterial Community between Host Twigs and *Lithosaphonecrus arcoverticus* Galls

**DOI:** 10.3390/insects12110982

**Published:** 2021-10-29

**Authors:** Xue-Mei Yang, Yu Hui, Lv-Quan Zhao, Dao-Hong Zhu, Yang Zeng, Xiao-Hui Yang

**Affiliations:** 1 Key Laboratory of Protein Chemistry and Developmental Biology of Fish of Education Ministry of China, State Key Laboratory of Developmental Biology of Freshwater Fish, College of Life Science, Hunan Normal University, Changsha 410081, China; xuemeiyang@hunnu.edu.cn (X.-M.Y.); yuhui@hunnu.edu.cn (Y.H.); 2 Co-Innovation Center for Sustainable Forestry in Southern China, College of Forestry, Nanjing Forestry University, Nanjing 210037, China; zhaolvquan@njfu.edu.cn; 3 Laboratory of Insect Behavior and Evolutionary Ecology, Central South University of Forestry and Technology, Changsha 410004, China; dhzhu@csuft.edu.cn (D.-H.Z.); t20162281@csuft.edu.cn (Y.Z.)

**Keywords:** insect galls, auxin, cytokinin, gall wasp, bacterial community, *Lithosaphonecrus arcoverticus*, *Lithocarpus glaber*

## Abstract

**Simple Summary:**

Insect galls are characterized by high concentrations of auxins and cytokinins. We calculated the correlation between the concentrations of indoleacetic acid (IAA), trans-zeatin riboside (tZR) and isopentenyladenine (iP) and the bacterial community structure of *Lithosaphonecrus arcoverticus* galls. Our results indicated the concentrations of IAA, tZR and iP were positively correlated with the bacterial community structure of *L. arcoverticus* galls. We suggest the high concentrations of IAA, tZR and iP may affect the bacterial community structure of *L. arcoverticus* galls.

**Abstract:**

Insect galls are the abnormal growth of plant tissues induced by a wide variety of galling insects and characterized by high concentrations of auxins and cytokinins. It remains unclear whether the auxins and cytokinins affect the bacterial community structure of insect galls. We determined the concentrations of indoleacetic acid (IAA) as an example of auxin, trans-zeatin riboside (tZR) and isopentenyladenine (iP) as cytokinins in *Lithosaphonecrus arcoverticus* (Hymenoptera: Cynipidae) galls and the galled twigs of *Lithocarpus glaber* (Fagaceae) using liquid chromatography–tandem mass spectrometry. Moreover, for the first time, we compared the bacterial community structure of *L. arcoverticus* galls and galled twigs by high-throughput sequencing, and calculated the Spearman correlation and associated degree of significance between the IAA, tZR and iP concentrations and the bacterial community structure. Our results indicated the concentrations of IAA, tZR and iP were higher in *L. arcoverticus* galls than in galled twigs, and positively correlated with the bacterial community structure of *L. arcoverticus* galls. We suggest the high concentrations of IAA, tZR and iP may affect the bacterial community structure of *L. arcoverticus* galls.

## 1. Introduction

Insect galls are abnormal plant tissues induced by a wide variety of galling insects such as gall wasps, gall midges, gall aphids, gall flies, gall moths, psyllids and thrips [1]. The morphology and structure of insect galls vary depending on the galling insects and host plants [2].

Insect galls are characterized by fast growth, tissue differentiation, cell hypertrophy and hyperplasia associated with high levels of auxins and cytokinins in the developmental period [3]. The phytohormones such as auxins are involved in plant cell elongation and proliferation, whereas cytokinins promote cell differentiation and proliferation [4]. Previous studies have indicated that the levels of auxins and cytokinins are higher in midge galls [5], aphid galls [6], fly galls [7], moth galls [8], psyllids galls [9,10] and sawfly galls [11] than in the un-galled tissues of host plants. Moreover, the enzyme immunoassays have shown a high content of cytokinin such as dihydrozeatin riboside in insect galls induced by the gall wasp *Dryocosmus kuriphilus* [12].

The primary sources of auxins and cytokinins in insect galls include the secretion of galling insects and endogenous synthesis. Auxins and cytokinins are abundant and widespread among galling insects, and galling insects may secrete auxins and cytokinins into insect galls [13,14,15,16]. Previous studies have indicated that many IAA-responsive and CK-responsive genes are upregulated in gall tissues [11,17,18,19,20,21]. Additionally, the insect galls are plant tissues and may synthesize IAA and CK. Furthermore, some bacteria can synthesize auxins and cytokinins, and regulate the growth and development of plants [4]. However, recent studies suggested the fast growth of gall induction is not consistently mediated by a bacterial symbiont or bacterial community [22].

Auxins and cytokinins have been demonstrated to play essential roles in bacterial growth and development [23]. For example, auxins can affect bacterial colonization and motility by regulating the gene expression of the flagellum [24]. Furthermore, several reports have shown that auxins and cytokinins participate in plant defense responses to pathogen infections [25]. Some studies have confirmed the differences of fungal community structure between the galled tissues of host plants and insect galls including cynipid galls [26,27], midge galls [28] and aphid galls [29]. To date, little information has been published on the differences of bacterial community structure between insect galls and the galled tissues of host plants. Moreover, whether the high contents of auxins and cytokinins affect the bacterial community structure of insect galls remains unclear.

The insect galls of *Lithosaphonecrus arcoverticus* (Hymenoptera: Cynipidae) grow rapidly on the galled twigs of *Lithocarpus glaber* in September and October [30]. In this study, we determined the contents of auxins such as indoleacetic acid (IAA), as well as cytokinins such as trans-zeatin riboside (tZR) and isopentenyladenine (iP) in *L. arcoverticus* galls and galled twigs by liquid chromatography–tandem mass spectrometry. We compared the bacterial community composition of *L. arcoverticus* galls and galled twigs using high-throughput sequencing. We explored the transmission of bacteria by the plant’s vascular system (vascular transmission) between *L. arcoverticus* galls and galled twigs, and the effects of the pathways of IAA, tZR and iP on the bacterial community structure of *L. arcoverticus* galls.

## 2. Materials and Methods

### 2.1. Sample Collection

*L. arcoverticus* galls and the galled twigs of *L. glaber* were collected simultaneously from eight trees at Fanling Town (28.41° N/113.31° E), China, in September 2020 (Figure S1). The samples were washed with sterile phosphate-buffered saline buffer for 30 s, and then were surface-sterilized with 70% ethanol for 2 min and 5% sodium hypochlorite (0.1% Tween 80) for 5 min, followed by washing five times with sterile water. All samples were flash-frozen for 15 min in liquid nitrogen. All frozen samples were transported to the laboratory on dry ice and stored at −80 °C until processing. The larvae of *L. arcoverticus* were removed from insect galls to avoid potential contamination. The sample size was eight for the *L. arcoverticus* and galled twig group in subsequent experiments including measurement of auxins and cytokinins, and the high-throughput sequencing of bacterial 16S ribosomal RNA.

### 2.2. Extraction and Measurement of Auxins and Cytokinins

Independent dilutions were made from methanol with 0.1% formic acid to prepare standard solutions of IAA, tZR and iP at concentrations of 0.1 ng/mL, 0.2 ng/mL, 0.5 ng/mL, 2 ng/mL, 5 ng/mL, 20 ng/mL, 50 ng/mL and 200 ng/mL. The standard samples of IAA, tZR and iP were purchased from Sigma-Aldrich (St. Louis, MO, USA).

For each sample of *L. arcoverticus* galls and galled twigs, 1 g tissue was pulverized in liquid nitrogen. A total of 10 mL isopropanol/hydrochloric acid extraction buffer was added into each sample and followed by shaking at 4 °C for 30 min; then 20 mL dichloromethane was added and followed by shaking at 4 °C for 30 min. The mixtures were centrifuged at 13,000× *g* for 10 min at 4 °C, and the lower organic phase was dried under N_2_ in the dark and dissolved in 400 μL methanol (0.1% formic acid). The collected solution was then filtered through a 0.22 μm filter membrane and used to detect the contents of IAA, tZR and iP.

The levels of IAA, tZR and iP in the *L. arcoverticus* galls and galled twigs were measured using an external standard method by high-performance liquid chromatography–tandem mass spectrometry (Agilent series 1290 system, Agilent Technologies, Santa Clara, CA, USA; QTrap6500 mass spectrometer, Ab Sciex, CA, USA). The chromatographic separation was achieved on a reversed phase liquid chromatography column (Poroshell120 SB-C18, 2.1 × 150 mm, 2.7 μM) at a column temperature of 30 °C. The mobile phase consisted of a mixture of solvent A (0.1% acetic acid in methanol) and solvent B (0.1% acetic acid in water) at a flow rate of 0.3 mL/min. The mass spectroscopy was conducted under positive electrospray ionization and multiple reaction monitoring mode. The conditions of mass spectrometry were as follows: the spray voltage was 4500 V; the pressures of the curtain gas, nebulizer gas and auxiliary gas were 15, 65 and 70 pounds per square inch, respectively; and the atomizing temperature was 400 °C. The selected reaction monitoring conditions for protonated or deprotonated auxins and cytokinins were as follows: the mass to charge (*m*/*z*) ratios of the mother ions of IAA, tZR and iP were 176.2, 352.3 and 204.1, respectively; the *m*/*z* of the son ions of IAA, tZR and iP were 129.8, 220.2 and 136.1, respectively; the declustering potentials of IAA, tZR and iP were 65, 90 and 80 V, respectively; the collision energies of IAA, tZR and iP were 12, 25 and 17 V, respectively. The measurements of IAA, tZR and iP were performed by Zoonbio Biotechnology Co. Ltd. (Nanjing, China).

### 2.3. DNA Extraction, PCR Amplification, Library Construction and High-Throughput Sequencing

Total DNA of *L. arcoverticus* galls and galled twigs was extracted and purified with an E.Z.N.A.^®^ soil DNA kit (Omega Bio-tek, Norcross, GA, USA). The V5–V7 region of the bacterial 16S ribosomal RNA was amplified using nested PCR primers with the first primer pair 799F (5′-AACMGGATTAGATACCCKG-3′)-1392R(5′-ACGGGCGGTGTGTRC-3′) and the second pair 799F (5′-AACMGGATTAGATACCCKG-3′)-1193R (5′-ACGTCATCCCCACCTTCC-3′). Extraction blanks were used with each batch of samples, and the negative controls were used in the 16S amplicon screening process to assess reagents and environmental contamination. Negative controls consisted of extraction blanks and sterile water. If some samples were contaminated, these contaminated samples were excluded from all the analysis. The cycling conditions of first-round nested PCR were 5 min at 95 °C, followed by 27 cycles of 30 s at 95 °C, 30 s at 53 °C, 45 s at 72 °C and a final elongation step of 15 min at 72 °C. The cycling conditions of second-round nested PCR were the same as those of the first-round nested PCR, except that 13 cycles were performed and 1 μL of the first-round PCR products was used as the templates. The amplification was performed using the GeneAmp PCR System 9700 (Applied Biosystems, London, UK) in a 20 μL reaction volume: 4 μL 5×TransStart FastPfu buffer, 0.4 μL Taq polymerase, 0.8 μL forward and reverse primer (5 μM), 2 μL dNTPs (2.5 mM each), 1 μL DNA template and 11 μL H_2_O. The PCR products were separated from 2% agarose gel, then were purified and quantified with a Quantus™ Fluorometer (Promega, Madison, WI, USA) and an AxyPrep DNA Gel Extraction Kit (Axygen Biosciences, Union City, CA, USA). Library preparation and high-throughput paired-end sequencing were performed by Majorbio Bio-Pharm Technology Co. Ltd. (Shanghai, China) using an Illumina MiSeq PE300 sequencing platform (Illumina, San Diego, CA, USA) and a NEXTFLEX Rapid DNA-Seq Kit (Bioo Scientific, Austin, TX, USA). The raw data have been deposited in the NCBI Sequence Read Archive (SRA) database and are available under the SRA accession number SRP334687.

### 2.4. Bioinformatics and Statistical Analysis

The raw sequencing reads of the 16S ribosomal RNA gene were quality-filtered with fastp software [31] and merged using FLASH software [32] according to the following criteria: sequence length > 200 bp, mean quality score ≥ 20 and no ambiguous bases. After quality filtering, high-quality reads were clustered into operational taxonomic units (OTUs) at a similarity cutoff value of 97% using UPARSE [33]. The representative sequence of each OTU was analyzed and annotated from the phylum to species level with RDP classifier version 2.4 [34] and the Silva database at a 0.8 confidence threshold for the molecular identification of bacteria. For each sample, 39,986 sequences were randomly selected to generate an OTU table that recorded the abundance and taxonomy of each OTU. The OTU table was used for the subsequent statistical analysis.

Statistical analysis was performed using R version 3.6.3 (https://www.r-project.org, 17 March 2021). Data of IAA, tZR and iP contents were approximately normally distributed, and the variance was not homogeneous between groups. *L. arcoverticus* galls and associated galled twigs on an individual tree. We used a two-tailed paired *t*-test to compare the difference of IAA, tZR and iP contents in *L. arcoverticus* galls and galled twigs. We counted the number of unique, common and high abundance bacteria of *L. arcoverticus* galls and galled twigs at the genus level. The bacterial genera with a relative abundance >1% were defined as high abundance genera. The Shannon index measures were used to evaluate the α-diversity of the bacterial community in *L. arcoverticus* galls and galled twigs at the genus level. The calculation of the Shannon index was based on an OTU table at the genus level and the Shannon formula (Formula S1 in Supplementary Materials). The Shannon index was tested for normal distribution (Shapiro–Wilk test) and homogeneity of variance (Bartlett’s test). The variance of the Shannon index was not homogeneous, and the Wilcoxon signed rank test was used to evaluate potential significant differences of Shannon index between *L. arcoverticus* galls and galled twigs.

Distance-based redundancy analysis (db-RDA) was performed to analyze the correlation between the IAA, tZR and iP contents and the bacterial community structure of *L. arcoverticus* galls and galled twigs at the genus level. First, the overall difference in community structure was assessed using permutational multivariate analysis of variance (PERMANOVA) based on the weighted UniFrac distance with 1000 permutations. Second, the bacterial community structures of *L. arcoverticus* galls and galled twigs at the genus level were compared using principal coordinate analyses based on the weighted UniFrac distance with the R package “ape” [35]. Third, the Spearman correlation and associated degree of significance between the IAA, tZR and iP contents and the bacterial community structure were calculated using the “capscale” and “envfit” functions in the R package “vegan” [36,37].

The linear discriminant analysis (LDA) effect size (LEfSe) (http://huttenhower.sph.harvard.edu/galaxy/, 7 April 2021) was used to reveal the dominant bacteria in *L. arcoverticus* galls and galled twigs from the phylum to the genus level. The dominant bacteria refer to those dominant bacterial taxa whose relative abundance was significantly higher than the other group. First, the Wilcoxon rank-sum test was used to detect those dominant bacterial taxa from the kingdom to the genus level. Then, LDA was used to calculate the effect size of each taxon; the higher the LDA score, the greater the influence of taxa on the difference. The LDA score threshold was set to four.

## 3. Results

### 3.1. Contents of IAA, tZR and iP in L. arcoverticus Galls and the Galled Twigs of L. glaber

**Figure 1:**
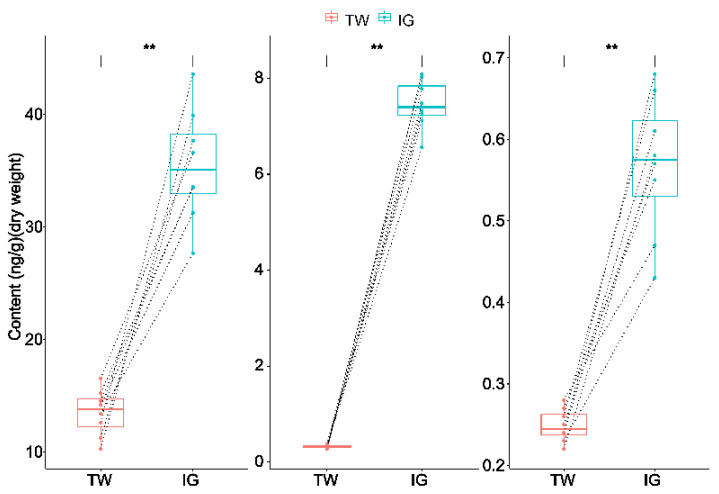
The contents of IAA, tZR and iP in *Lithosaphonecrus arcoverticus* galls and the galled twigs of *Lithocarpus glaber*. IAA, tZR and iP represent indoleacetic acid, trans-zeatin riboside and isopentenyladenine, respectively. IG and TW indicate *L. arcoverticus* galls and the galled twigs of *L. glaber*, respectively. Dotted lines indicate galled twigs and paired *L. arcoverticus* galls. The top and bottom horizontal lines of the boxplot indicate 25th and 75th percentiles, respectively. The lines within the box indicate median values. ** indicates a significant difference (*p* < 0.01).

The contents of IAA, tZR and iP in *L. arcoverticus* galls were significantly higher than that in the galled twigs of *L. glaber* (paired *t*-test, t = 16.56 for IAA; t = 39.69 for tZR; t = 9.45 for iP; df = 7 and *p* < 0.01 for all cases) (Figure 1).

### 3.2. Correlation between IAA, tZR and iP Contents and the Bacterial Community Structure of L. arcoverticus Galls and the Galled Twigs of L. glaber

**Figure 2:**
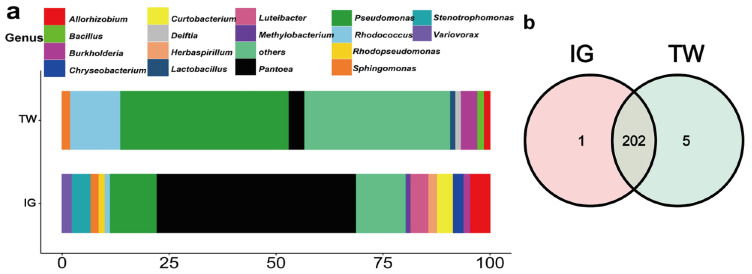
The community composition of bacteria at the genus level in *Lithosaphonecrus arcoverticus* galls and the galled twigs of *Lithocarpus glaber*. IG and TW indicate *L. arcoverticus* galls and the galled twigs of *L. glaber*, respectively. (**a**) The relative abundance of high abundance genera. The horizontal coordinate indicates the relative abundance of the high abundance genera. The bacterial genera with a relative abundance above 1% are defined as high abundance genera. Bacterial genera with a relative abundance below 1% are grouped as “others”. (**b**) The Venn diagram of the community composition of bacteria in *L. arcoverticus* galls and galled twigs. The number shows the number of bacterial genera unique or common to *L. arcoverticus* galls and galled twigs.

A total of 16 phyla, 31 classes, 73 orders, 118 families, 208 genera, 329 species and 459 OTUs were found in the bacterial community of *L. arcoverticus* galls and the galled twigs of *L. glaber* (Table 1). From the phylum to OTU level, the numbers of bacteria in *L. arcoverticus* galls were less than those in galled twigs (Table 1). We identified 14 and nine high abundance genera of bacteria in *L. arcoverticus* galls and galled twigs, respectively (Figure 2a). The *Pantoea* genus had the highest relative abundance (46.81%) in *L. arcoverticus* galls, and the *Pseudomonas* genus had the highest relative abundance (39.37%) in the galled twigs of *L. glaber* (Figure 2a). Furthermore, a total of 202 genera were common to *L. arcoverticus* galls and galled twigs, and the numbers of unique bacterial genera of *L. arcoverticus* galls and galled twigs were one and five, respectively (Figure 2b).

**Figure 3:**
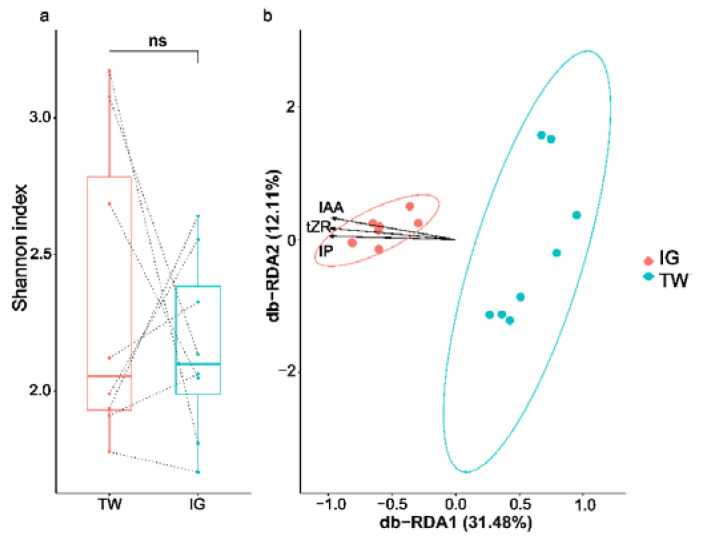
The IAA, tZR, iP and bacterial community structure at the genus level of *Lithosaphonecrus arcoverticus* galls and the galled twigs of *Lithocarpus glaber*. IG and TW indicate *L. arcoverticus* galls and the galled twigs of *L. glaber*, respectively. (**a**) Boxplot of bacterial α-diversity at the genus level of *L. arcoverticus* galls and galled twigs, as measured by the Shannon index. NS indicates that any difference is not significant. Dotted lines indicate galled twigs and paired *L. arcoverticus* galls. The top and bottom horizontal lines of the boxplot indicate 25th and 75th percentiles, respectively. The lines within the box indicate median values. (**b**) Distance-based redundancy analysis (db-RDA) between IAA, tZR and iP contents and the bacterial community structure of *L. arcoverticus* galls and galled twigs based on the weighted UniFrac distance. Each solid point represents the bacterial community from an individual specimen. The horizontal and vertical axes show the first and second redundancy analysis coordinates (db-RDA1 and db-RDA2), respectively. The percentage shows the proportion of the total variation explained by each axis. The ellipse indicates the 95% confidence interval around the centroid for *L. arcoverticus* galls and galled twigs. IAA, tZR and iP represent indoleacetic acid, trans-zeatin riboside and isopentenyladenine, respectively. The length of the straight line arrow represents the magnitude of the effects of IAA, tZR and iP contents on the bacterial community structure of *L. arcoverticus* galls and galled twigs. The angles between straight line arrow and solid point indicate the correlation between IAA, tZR and iP contents and the bacterial community structure of *L. arcoverticus* galls and galled twigs, and the acute and obtuse angles indicate a positive and negative association, respectively.

The α-diversity did not significantly differ between *L. arcoverticus* galls and the galled twigs of *L. glaber* (Wilcoxon signed rank test, v = 21, *p* = 0.67) (Figure 3a), whereas significant differences were observed between the bacterial community structure of *L. arcoverticus* galls and the galled twigs of *L. glaber* (PERMANOVA, r^2^ = 0.39, *p* < 0.01). The bacterial community structure of *L. arcoverticus* galls was clearly different from that of the galled twigs of *L. glaber* (Figure 3b). Moreover, Envfit and db-RDA analyses showed a significant positive correlation between the contents of IAA (r^2^ = 0.81, *p* < 0.01), tZR (r^2^ = 0.71, *p* < 0.01) and iP (r^2^ = 0.83, *p* < 0.01) and the bacterial community structure of *L. arcoverticus* galls (Figure 3b).

### 3.3. Dominant Bacteria of L. arcoverticus Galls and the Galled Twigs of L. glaber

**Figure 4:**
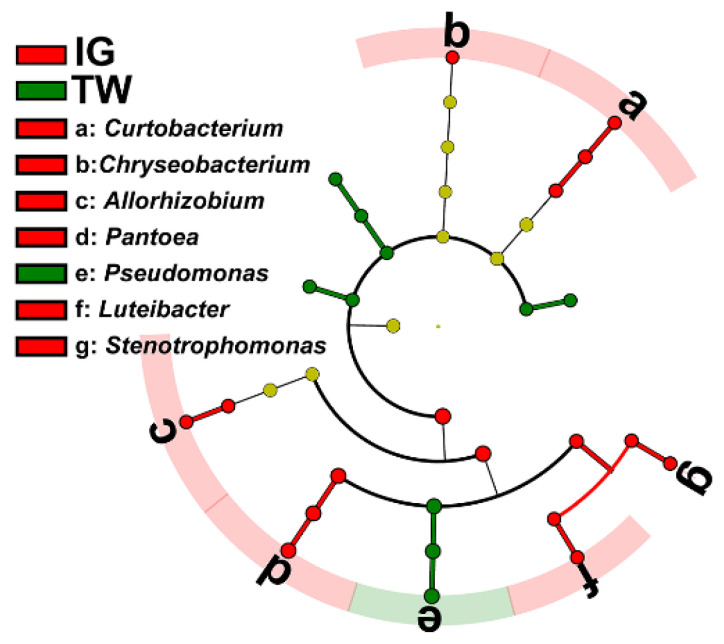
The dominant genera of bacteria in *Lithosaphonecrus arcoverticus* galls and the galled twigs of *Lithocarpus glaber*. IG and TW indicate *L. arcoverticus* galls and the galled twigs of *L. glaber*. The LEfSe plot of the dominant bacteria in *L. arcoverticus* galls and galled twigs. The cladogram levels, from the inner to outer rings, stand for kingdom, phylum, class, order, family and genus. The red and green nodes of the cladogram show the dominant bacteria of *L. arcoverticus* galls and galled twigs from the kingdom to genus level, respectively. The yellow nodes show the nondominant bacteria in *L. arcoverticus* galls and galled twigs. The letters from a to g represent dominant bacterial genera in *L. arcoverticus* galls and galled twigs.

The LEfSe analysis showed that one phylum, one class, three orders, five families and six genera were dominant in the bacterial community of *L. arcoverticus* galls, whereas three phyla, three classes, two orders, one family and one genus were dominant in the bacterial community of galled twigs (Figure 4).

## 4. Discussion

### 4.1. Vascular Transmission of Bacteria between L. arcoverticus Galls and the Galled Twigs of L. glaber

The *L. arcoverticus* galls and galled twigs shared most genera of the bacterial community. These findings suggested a potential possibility that the bacteria might transmit between *L. arcoverticus* galls and galled twigs through the plant’s vascular system. We suggest that structural connections and transport of substances may be associated with the potential vascular transmission between *L. arcoverticus* galls and galled twigs. The vascular bundles of *L. arcoverticus* galls connect with the vascular system of host plants [30]. This structural connection may be beneficial for the vascular transmission of bacteria between *L. arcoverticus* galls and galled twigs. For example, plant endophytes can invade adjacent plant tissues by secreting virulence factors such as extracellular polysaccharides and plant cell wall degrading enzymes [38]. Furthermore, water and nutrients can be transported from host plants to cynipid galls via vessels and sieve tubes, respectively [2]. Previous studies have confirmed the transmission of bacteria through the vascular system intended for substance transportation, using green fluorescent protein labeling and β-glucuronidase staining [39,40]. For example, the species of *Allorhizobium* [41] and *Pantoea* [42] genera in the soil can colonize the root tissues and then migrate from root to leaf through the vascular system for substance transportation. The *Allorhizobium* and *Pantoea* genera were shared by *L. arcoverticus* galls and galled twigs. Thus, we suggest that the transport of substances may favor the bacterial transmission between *L. arcoverticus* galls and galled twigs.

### 4.2. The Potential Effect of Auxins and Cytokinins on the Bacterial Community Structure of L. arcoverticus Galls

The differences in bacterial community structure between the insect galls and the galled twigs may be associated with multiple factors including the form of the gall, the differences in the surface texture and chemical composition between insect galls and the galled twigs. The differences in bacterial community structure between *L. arcoverticus* galls and galled twigs may be associated with the differences of auxin and cytokinin content. For example, the auxins and cytokinins may affect the bacteria of *L. arcoverticus* galls in a variety of ways [4]. First, auxins and cytokinins are important signaling molecules that directly affect bacterial physiology and adaptation to varying environments [24,43]. For example, exogenous IAA can result in the upregulation of environmental stress-related genes of *Bradyrhizobium japonicum*, such as heat shock proteins, cold shock protein and exopolysaccharide genes [44]. Thus, the high IAA, tZR and iP contents may impose direct and special influence on the growth and development of bacteria in *L. arcoverticus* galls. Second, auxins and cytokinins can mediate nutrient metabolism in plant tissues [45,46]. The high contents of IAA, tZR and iP may alter the levels and composition of nutrients in *L. arcoverticus* galls, thus providing unique carbon and nitrogen sources for the bacterial community in *L. arcoverticus* galls. In fact, the levels and composition of carbohydrates [47], lipids [48,49], protein [50,51] and free amino acids [52] in cynipid galls differ from those in adjacent galled tissues. Finally, the auxins and cytokinins are involved in the plant’s defense against pathogens through communicating with jasmonic acid and salicylic acid signaling pathways [53,54,55]. The phytohormones jasmonic acid and salicylic acid are the primary regulators of plant responses to attacks by pathogens, and they affect the activity of defense-related enzymes and the production of secondary metabolites [55]. For example, jasmonic acid and salicylic acid are associated with tannins and reactive oxygen species [56], and high levels of tannin and reactive oxygen species can inhibit the growth of some bacteria [57,58]. Previous studies have indicated high levels of tannin [59,60], polyphenol oxidase [48,49,61] and reactive oxygen species [62,63] in cynipid galls. Therefore, we speculated that the high contents of IAA, tZR and iP might participate in plant defense and provide a particular habitat for the bacteria of *L. arcoverticus* galls.

## 5. Conclusions

In conclusion, this study indicated that *L. arcoverticus* galls and the galled twigs of *L. glaber* were generally inhabited by the same genera but the proportions between these genera were different, and the concentrations of IAA, tZR and iP were higher in *L. arcoverticus* galls than in galled twigs. This study also provided the first evidence that the concentrations of IAA, tZR and iP were positively correlated with the bacterial community structure of *L. arcoverticus* galls.

We suggest that structural connections and transport of substances may be associated with the potential vascular transmission between *L. arcoverticus* galls and galled twigs. Furthermore, we suggest the auxins and cytokinins may affect the bacteria of *L. arcoverticus* galls in a variety of ways including affecting bacterial physiology and adaptation, mediating nutrients metabolism in plant tissues and participating in plant defense.

## Figures and Tables

**Table 1:** The total number of bacteria in *Lithosaphonecrus arcoverticus* galls and the galled twigs of *Lithocarpus glaber* at different taxon levels.

	Phylum	Class	Order	Family	Genus	Species	OTU
*L. arcoverticus* galls	15	30	71	114	203	323	439
Galled twigs	16	31	73	118	207	327	452
Total	16	31	73	118	208	329	459

## Data Availability

The raw data have been deposited in the NCBI Sequence Read Archive (SRA) database and are available under the SRA accession number SRP334687.

## Supplementary Materials

The following are available online at https://www.mdpi.com/article/10.3390/insects12110982/s1, Figure S1: The *Lithosaphonecrus arcoverticus* galls and the galled twigs of *Lithocarpus glaber*. Formula S1: The formula of Shannon index based on OTU table.
