# Supplementary material for: Comparison of Auxin and Cytokinins Concentrations, and the Structure of Bacterial Community between Host Twigs and Lithosaphonecrus arcoverticus Galls
Source: Insects. 2021 Oct 29;12(11):982. doi: 10.3390/insects12110982 (PMC8618787; doi:10.3390/insects12110982)
Supplement: Supplementary file 1 [file insects-12-00982-s001.zip › Formula S1.pdf]

$$H_{shannon} = -\sum_{i=1}^{S_{obs}} \frac{n_i}{N} ln \frac{n_i}{N}$$

N refers to total number of sequences of each sample.

n_i refers to the number of sequences of the i-th OUT.

S_{obs} refers to actual number of OTU.

ln denotes natural logarithm and Σ denotes summation.
