# Supplementary figures and images for: Comparison of Auxin and Cytokinins Concentrations, and the Structure of Bacterial Community between Host Twigs and Lithosaphonecrus arcoverticus Galls
Source: Insects. 2021 Oct 29;12(11):982. doi: 10.3390/insects12110982 (PMC8618787; doi:10.3390/insects12110982)

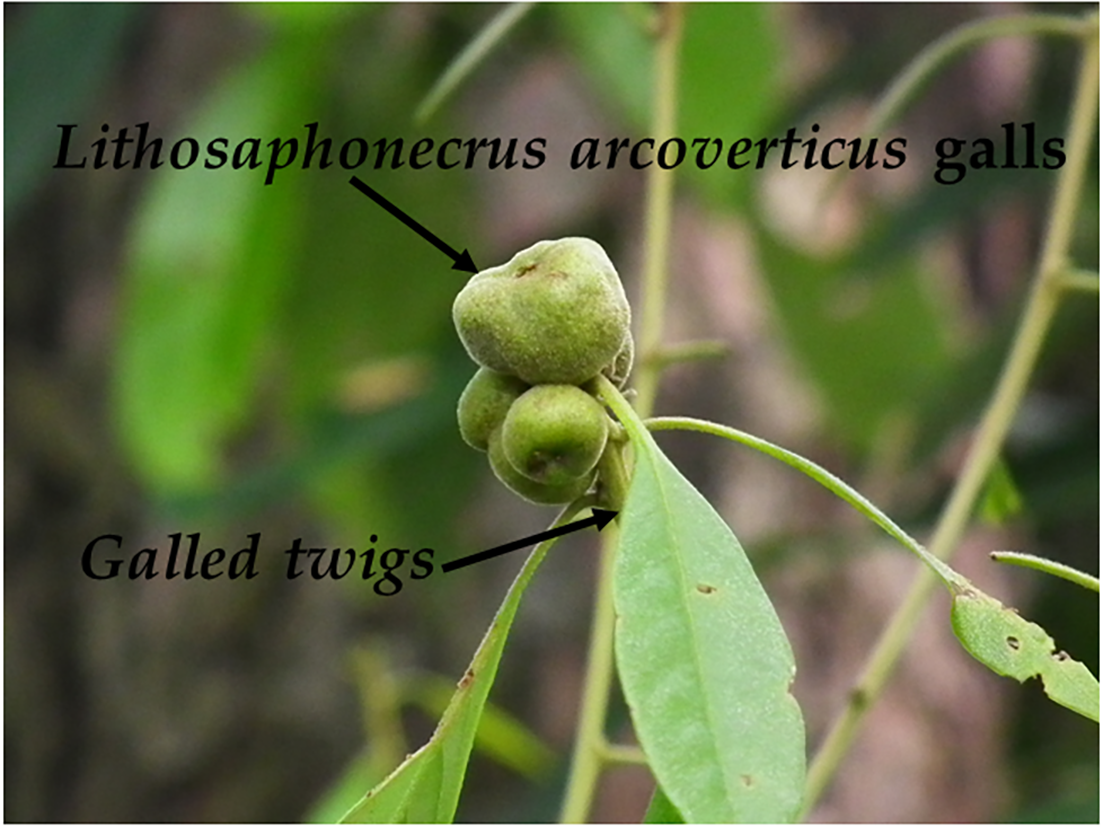

Supplement: Supplementary file 1 [file insects-12-00982-s001.zip › Figure S1.tif]
